# GATA2 rs2335052 Polymorphism Predicts the Survival of Patients with Colorectal Cancer

**DOI:** 10.1371/journal.pone.0136020

**Published:** 2015-08-19

**Authors:** Xijuan Liu, Beihai Jiang, Aidong Wang, Jiabo Di, Zaozao Wang, Lei Chen, Xiangqian Su

**Affiliations:** 1 Key laboratory of Carcinogenesis and Translational Research (Ministry of Education), Central Laboratory, Peking University Cancer Hospital & Institute, Beijing, China; 2 Key laboratory of Carcinogenesis and Translational Research (Ministry of Education), Department of Minimally Invasive Gastrointestinal Surgery, Peking University Cancer Hospital & Institute, Beijing, China; Icahn School of Medicine at Mount Sinai, UNITED STATES

## Abstract

**Background:**

GATA binding protein 2 (GATA2) is a transcription factor that has essential roles in hematologic malignancies and progression of various solid tumors. Our previous studies suggested that high GATA2 expression is associated with recurrence of colorectal cancer (CRC). However, the influence of GATA2 single nucleotide polymorphisms (SNPs) on the survival of CRC remains unknown.

**Methods:**

We genotyped GATA2 SNP rs2335052 using Sanger sequencing after PCR amplification, and determined GATA2 expression by immunohistochemistry in a cohort of 180 CRC patients. Kaplan-Meier survival analysis and Cox proportional hazard regression were used to analyze the association between the GATA2 rs2335052 genotypes and the clinical outcome of CRC.

**Results:**

We found that there was no significant correlation between the rs2335052 genotypes and the expression of GATA2. However, the Kaplan-Meier survival analysis suggested that the carriers of the A-allele of SNP rs2335052 were significantly associated with increased risk of recurrence and reduced disease-free survival (DFS), compared with those carrying the variant genotype of GG in rs2335052 (*P* = 0.021). Moreover, univariate and multivariate Cox regression analyses revealed that GATA2 SNP rs2335052 was an independent risk factor for the DFS of CRC patients.

**Conclusion:**

Our results demonstrated that GATA2 SNP rs2335052 is an independent predictor for prognosis of CRC patients. This raised the possibility that SNP rs2335052 may serve as a potential indicator for predicting recurrence of CRC after curative colectomy.

## Introduction

Colorectal cancer (CRC) is the third most prevalent cancer in the world and the second most common cause of cancer related death in the Western countries [1,2]. Data from the World Health Organization (WHO) shows that the incidence of CRC is rapidly increasing in many Asian countries including China [3]. Despite improvements in diagnosis, surgery, chemotherapy, and targeted therapy, the 5-year relative survival rate for patients with CRC still remains poor [4]. At present, the TNM staging system maintained by the American Joint Committee on Cancer (AJCC) is the most widely used guideline for staging and survival prediction [5]. However, the clinical outcomes of patients with CRC may vary considerably even within the same tumor stage, particularly of patients with stage II disease [6, 7]. Therefore, new molecular prognostic markers which could precisely stratify patients into different risk categories are clearly warranted.

GATA2 binding protein 2 (GATA2) is a zinc-finger transcription factor, which plays a crucial role in regulating transcription of genes involved in proliferation, development, and differentiation of hematopoietic cells [8–11]. There is growing evidence that altered GATA2 expression and constitutive heterozygous GATA2 mutation are associated with hematologic malignancies, as well as the development and progression of various solid tumors [12–20]. In sporadic and familial myeloid malignancies, high GATA2 expression and acquired or inherited mutations of the GATA2 gene have been reported [12–15]. For solid tumors, high levels of GATA2 expression are associated with poor prognosis and disease recurrence in prostate and CRC [16–18]. In addition, high GATA2 expression in non-cultured human breast carcinomas promotes the proliferation of breast cancer cells by inhibiting the transcription of PTEN [19]. However, Li et al reported that GATA2 expression was decreased in hepatocellular carcinoma tissues, and reduced expression of GATA2 correlated with poor prognosis of hepatocellular carcinoma [20]. These findings suggest that GATA2 may serve as a new predictor for the prognosis of different types of cancer.

GATA2 gene is located at chromosome 3q21. Many single nucleotide polymorphisms (SNPs) have been identified in human GATA2 gene, including rs2335052, rs3803, rs2713604, and so on [20–24]. These genetic variants may contribute to the altered expression and function of GATA2, and consequently, may have great influence on the clinical outcomes of CRC. However, the clinical significance of GATA2 SNPs in CRC has not been investigated yet.

In this study, we examined the SNP rs2335052 and the expression of GATA2 in a Chinese CRC cohort, and investigated the role of the GATA2 SNP rs2335052 as a predictor of the clinical outcome for CRC patients. Our results showed that although no significant correlation was observed between the rs2335052 genotypes and the expression of GATA2, the carriers of the A-allele of SNP rs2335052 showed a significant increased risk of recurrence and reduced disease-free survival (DFS). Thus, the GATA2 SNP rs2335052 may serve as a novel indicator for the prognosis of patients with CRC.

## Materials and Methods

### Ethics Statement

The study was approved and supervised by the research ethics committee of Peking University Cancer Hospital & Institute. Beijing, China. Written Informed Consents were obtained from all patients before surgery for general research using of their clinical data and specimens.

### Patients

This retrospective study included 180 patients who were undergone colectomy at the Department of Minimally Invasive Gastrointestinal Surgery, and histologically diagnosed with CRC at the Department of Pathology at Peking University Cancer Hospital & Institute from April 2009 to December 2011. All tumors were graded by two experienced pathologists according to the American Joint Committee on Cancer (AJCC) classification guidelines. Patients treated with pre-operative radiotherapy or adjuvant chemotherapy were excluded. Sixteen patients with pathologically confirmed metastasis or died of complications were also excluded. Formalin-fixed paraffin embedded (FFPE) tissue samples of patients were available for the study. DFS was calculated as the time from cancer diagnosis to either relapse or death from the last follow up.

### GATA2 SNP rs2335052 analysis

Genomic DNA was extracted from paraffin-embedded tumor specimens using FFPE DNA Kit (OMEGA, US) according to the manufacturer’s instructions. The GATA2 rs2335052 genotypes were determined by Sanger sequencing after amplification with polymerase chain reaction (PCR). The exon 3 of the GATA2 was amplified using the following primers: forward 5’-CCACCCTGATCCTCTCTCTCTTT and reverse 5’-TCACAGCTCCCCACCACAA. PCR was carried out in a 30μl volume, comprising 10–30ng genomic DNA, 0.2 mM dNTP, 1×PCR buffer, 10mM each primers, and 0.75 unit GoTaq DNA polymerase (Promega Cor., WI, USA) under the following amplification conditions: 2min at 95°C, followed by 35 cycles of 95°C for 30s, 60°C for 30 s, 72°C for 45 s. The PCR products were visualized on a 2% agarose gel and then subjected to direct sequencing. All fragments were sequenced using the ABI 3730XL Avant Genetic Analyzer (Applied Biosystems Inc., CA, USA). Finally, the sequencing results were analyzed with the Chromas software under the condition of signal/noise > 98%.

### GATA2 immunohistochemistry (IHC)

IHC analysis for GATA2 was performed using a primary rabbit polyclonal antibody against GATA2 (1:100, Santa Cruz sc-9008), followed by incubation with a secondary antibody from the EnVision kit (Dako Cytomation). The visualization signal was developed with diaminobenzidine (Sigma). Sections were counterstained with hematoxylin. The staining intensity was scored as follows [18, 25]:-, no staining or <10% positive cells; +, 10–20% weakly to moderately positive cells; ++, 10–20% intensively positive cells or 20–50% weakly positive cells; and +++, 20–50% positive cells with moderate to strong reactivity or >50% positive cells. The results reported by the two pathologists were highly consistent, and all disagreements were resolved by consensus after joint review. The score for GATA2 staining were described as either “low” or “high”, where “high” includes “++” and “+++” staining.

### Statistical analysis

The association between GATA2 SNP rs2335052 and the clinicopathological characteristics were analyzed with Chi-squared test. Survival was determined using the Kaplan-Meier methods and compared with the log-rank test. Univariate and multivariate survival analyses were performed using the Cox proportional hazard regression model. Fifteen patients lost to follow up were excluded in the Kaplan-Meier curve, univariate and multivariate analyses. The statistical analyses were performed with the software package SPSS 13.0 (SPSS, Inc., Chicago, IL, U.S.). *P* value of less than 0.05 was considered statistically significant.

## Results

### Patient characteristics and genotyping

**Fig 1:**
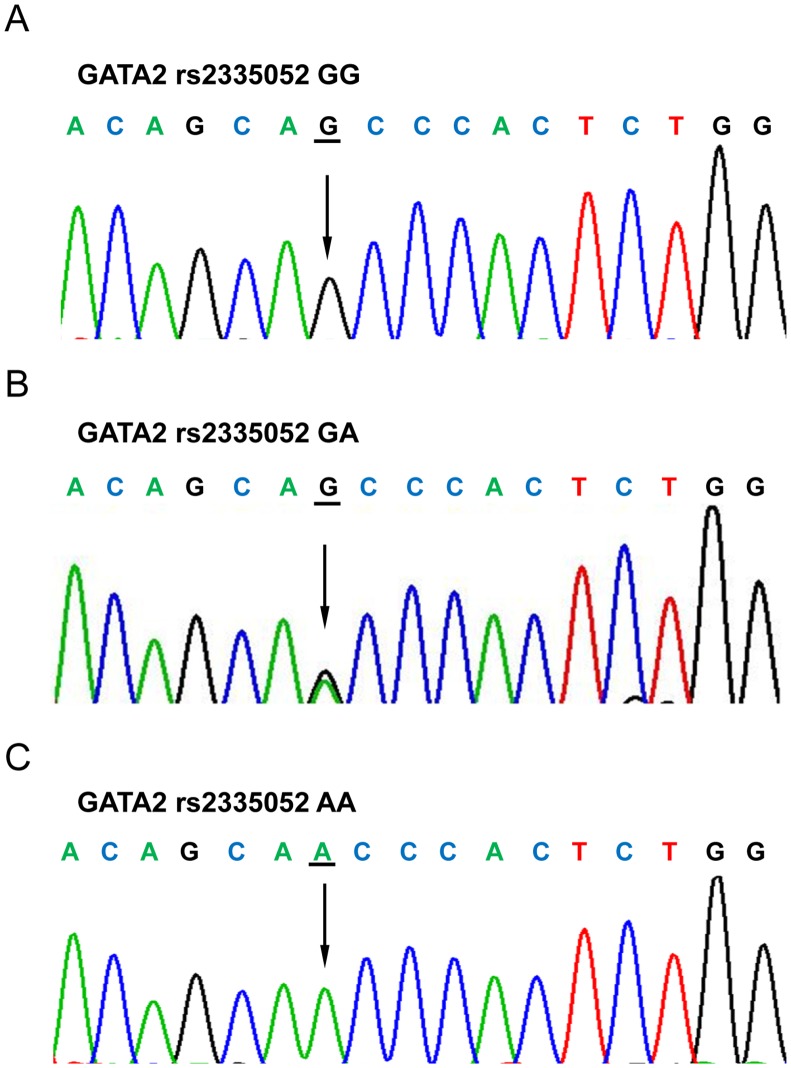
Sequencing results of GATA2 rs2335052 genotypes.

A total of 180 samples from patients with CRC were analyzed. Median follow up time in the cohort was 69.0 months. Carriers of the rs2335052 GG, GA, and AA genotypes were 64 (35.6%), 81 (45.0%), and 35 (19.4%), respectively (Fig 1). Details of the clinical characteristics and GATA2 rs2335052 genotypes are shown in Table 1. GATA2 SNP rs2335052 was significantly associated with disease-free survival (DFS) of CRC (*P* = 0.033). However, no significant associations were observed between rs2335052 genotypes and other clinicopathological characteristics (all *P*-values > 0.05).

**Table 1:** Clinicopathological features with respect to determined genotypes of included GATA2 SNP rs2335052.

Characteristic		Cases (%)	GA (%)	GG (%)	AA (%)	*P*-value
Age	<60 yr	77 (42.8)	40 (49.4)	23 (35.9)	14 (40.0)	0.249
	≥60 yr	103 (57.2)	41 (50.6)	41 (64.1)	21 (60.0)	
Gender	Female	78 (43.3)	30 (37.0)	35 (54.7)	13 (37.1)	0.074
	Male	102 (56.7)	51 (63.0)	29 (45.3)	22 (62.9)	
Tumor location	Colon	138 (76.7)	65 (80.2)	48 (75.0)	25 (71.4)	0.544
	Rectum	42 (23.3)	16 (19.8)	16 (25.0)	10 (28.6)	
Tumor size	≤4cm	90 (50.3)	44 (55.0)	30 (46.9)	16 (45.7)	0.522
	>4cm	89 (49.7)	36 (45.0)	34 (53.1)	19 (54.3)	
	Unknown	1				
Depth of invasion	T1/T2	18 (10.0)	9 (11.1)	7 (10.9)	2 (5.7)	0.641
	T3/T4	162 (90.0)	72 (88.9)	57 (89.1)	33 (94.3)	
Lymph node involvement	Negative	103 (57.2)	48 (59.3)	39 (60.9)	16 (45.7)	0.302
	Positive	77 (42.8)	33 (40.7)	25 (39.1)	19 (54.3)	
AJCC Stage	I/II	98 (54.4)	45 (55.6)	38 (59.4)	15 (42.9)	0.278
	III/IV	82 (45.6)	36 (44.4)	26 (40.6)	20 (57.1)	
Histological type	Adenocarcinoma	168 (93.3)	78 (96.3)	59 (92.2)	31 (88.6)	0.279
	Mucinous	12 (6.7)	3 (3.7)	5 (7,8)	4 (11.4)	
Tumor differentiation	Well	8 (4.8)	4 (5.2)	1 (1.7)	3 (10.0)	0.183
	Moderate	150 (90.4)	67 (87.0)	56 (94.9)	27 (90.0)	
	Poor	8 (4.8)	6 (7.8)	2 (3.4)	0 (0.0)	
	Unknown	14				
Chemotherapy	Not received	93 (51.7)	40 (49.4)	36 (56.3)	17 (48.6)	0.656
	Received	87 (48.3)	41 (50.6)	28 (43.8)	18 (51.4)	
Disease-free Survival	Negative	127 (76.5)	53 (68.8)	51 (87.9)	23 (74.2)	0.033
	Positive	39 (23.5)	24 (31.2)	7 (12.1)	8 (25.8)	
	Unknown	14				
Overall Survival	Alive	111 (67.3)	52 (68.4)	42 (72.4)	17 (54.8)	0.232
	Dead	54 (32.7)	24 (31.6)	16 (27.6)	14 (45.2)	
	Unknown	15				
GATA2 expression	Low	71 (43.3)	29 (39.7)	29 (49.2)	13 (40.6)	0.523
	High	93 (56.7)	44 (60.3)	30 (50.8)	19 (59.4)	
	Unknown	16				

Differences in categorical study variables between genotypes were tested for statistical significance with the Chi-squared test. Tumors were classified according to the guidelines of the American Joint Committee on Cancer (AJCC) staging system.

### Association of GATA2 rs2335052 genotypes with DFS

**Fig 2:**
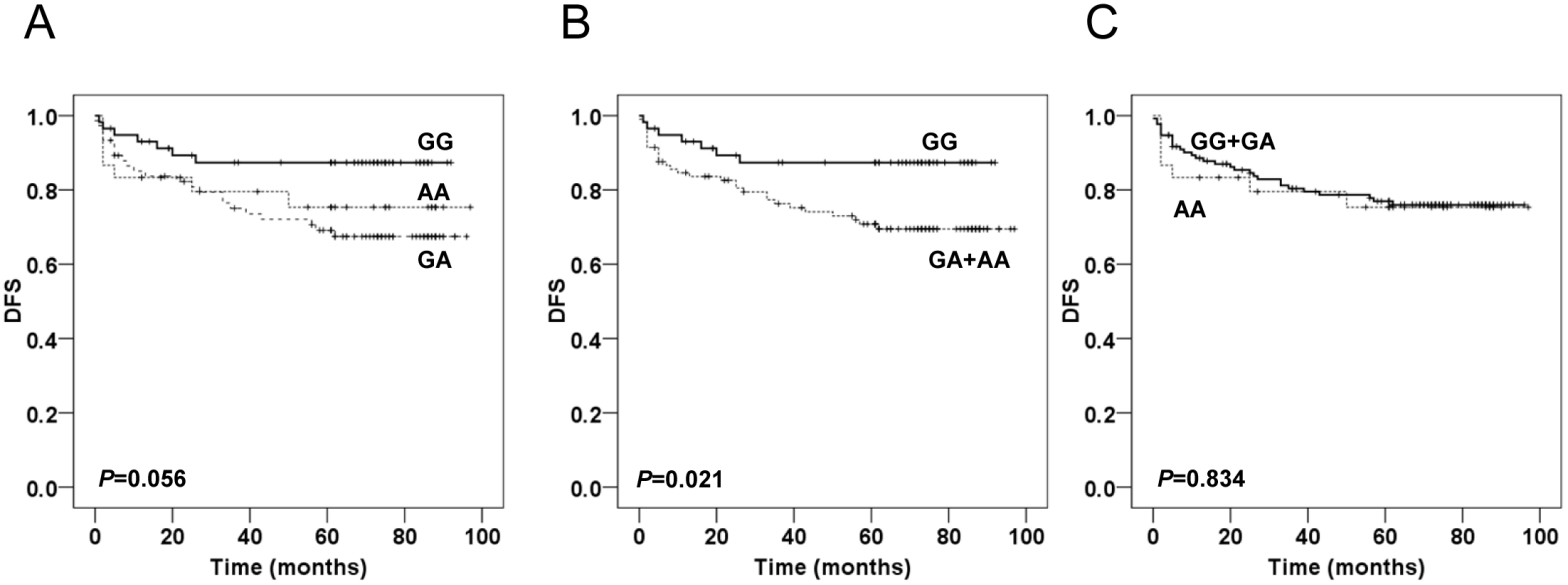
Kaplan-Meier analysis of DFS according to GATA2 SNP rs2335052. Kaplan-Meier curves are shown for an additive **(A)**, dominant **(B)**, and recessive model **(C)** of inheritance. The log-rank test was used to calculate *P* values.

Kaplan-Meier curves for DFS were graphically shown according to an additive, dominant, and recessive model of inheritance (Fig 2). The results revealed that SNP rs2335052 was significantly associated with DFS under the dominant model of inheritance (*P* = 0.021), which suggested that carriers of the A-allele of SNP rs2335052 (GA, AA) had a significantly increased risk of recurrence and reduced DFS (Fig 2B). The 5-year DFS rate of patients with carriers of the rs2335052 GA and AA genotypes was 69.0%, but for patients with carrier of the rs2335052 GG genotype, the rate was 92.0%. However, significant difference in DFS was not observed among GATA2 rs2335052 genotypes under the additive or recessive model (*P* = 0.056, *P* = 0.834, respectively, Fig 2A and 2C).

**Fig 3:**
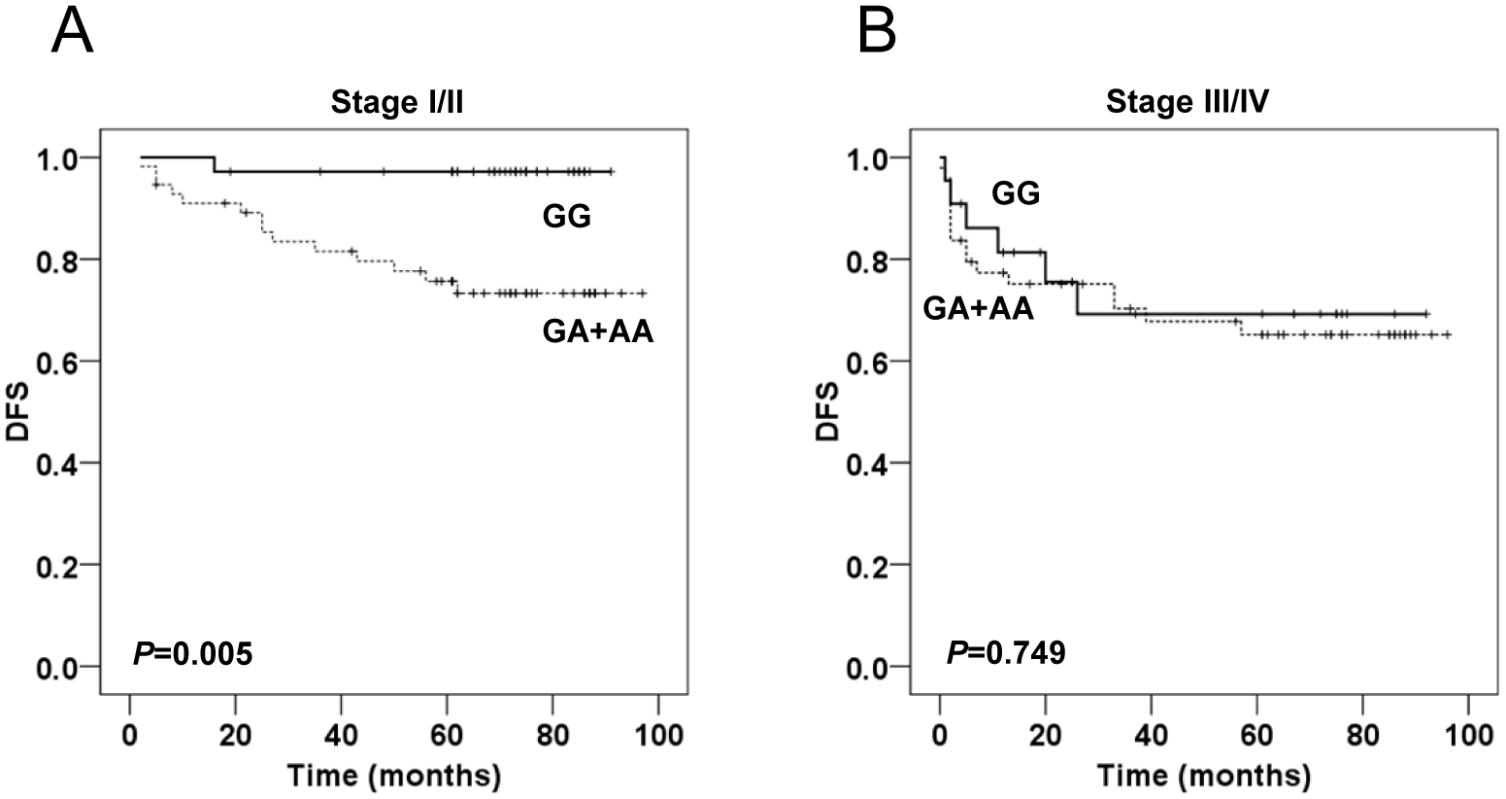
Kaplan-Meier analysis for DFS according to rs2335052 genotypes in CRC patients stratified by clinical stage. Kaplan-Meier curves indicated DFS for the subgroup of patients with stage I/II **(A)**, and stage III/IV **(B)** CRC. The log-rank test was used to calculate *P* values.

Additionally, we carried out a stratified analysis by the TNM stage (I/II and III/IV), to determine the prognostic significance of GATA2 rs2335052 genotypes in different tumor stages. Harboring rs2335052 GA or AA confirmed an adverse impact on DFS of stage I/II patients (*P* = 0.005, Fig 3A). However, the GATA2 rs2335052 genotypes were not correlated with prognosis of patients with stage III/IV diseases (*P* = 0.749, Fig 3B).

The prognostic value of GATA2 rs2335052 genotypes in DFS was evaluated by univariate and multivariate Cox regression analyses using a dominant model of inheritance. As shown in Table 2, in both univariate and multivariate analyses, patients with the A-allele of SNP rs2335052 (GA, AA) had a shorter period of DFS, suggesting a higher risk of recurrence (HR, 2.530; 95% CI, 1.111–5.761; *P* = 0.027 and HR, 2.825; 95% CI, 1.095–7.290; *P* = 0.032, for univariate and multivariate analysis, respectively), compared with the patients with the SNP rs2335052 GG genotype. The results suggest that the GATA2 SNP rs2335052 was an independent prognostic factor for disease recurrence. The relative risk of recurrence in the patients with rs2335052 GA and AA genotypes was 2.825-fold higher than that of the patients with the rs2335052 GG genotype, according to the multivariate analysis. Unfavorable survival outcomes were also significantly associated with male patients, stage III/IV CRC and poor or moderate tumor differentiation, based on univariate analysis (Table 2).

**Table 2:** Univariate and multivariate analyses of GATA2 rs2335052 genotypes in CRC patients with respect to DFS.

Variables	Univariate			Multivariate		
	HR	95%CI	*P*	HR	95%CI	*P*
Age (≥60 yr vs <60 yr)	1.112	0.580–2.132	0.749	0.986	0.483–2.012	0.969
Gender (Male vs Female)	2.437	1.149–5.166	**0.020**	1.957	0.888–4.316	0.096
Tumor location (Rectum vs Colon)	1.016	0.479–2.154	0.966	1.500	0.658–3.421	0.335
Tumor size (>4cm vs ≤4cm)	1.215	0.636–2.320	0.555	0.999	0.485–2.060	0.998
TNM stage (III/IV vs I/II)	2.381	1.233–4.597	**0.010**	2.232	1.051–4.741	0.037
Tumor differentiation (Poor/moderate vs Well)	4.294	2.921–6.312	**<0.0001**	5.821	3.470–9.764	**<0.0001**
Chemotherapy (Received vs Not received)	0.850	0.446–1.621	0.622	1.366	0.607–3.072	0.451
GATA2 SNP (GA/AA vs GG)	2.530	1.111–5.761	**0.027**	2.825	1.095–7.290	**0.032**

*HR* hazard ratio, *CI* confidence interval, *P* values in bold were statistically significant.

### Association between GATA2 rs2335052 genotypes and GATA2 expression

**Fig 4:**
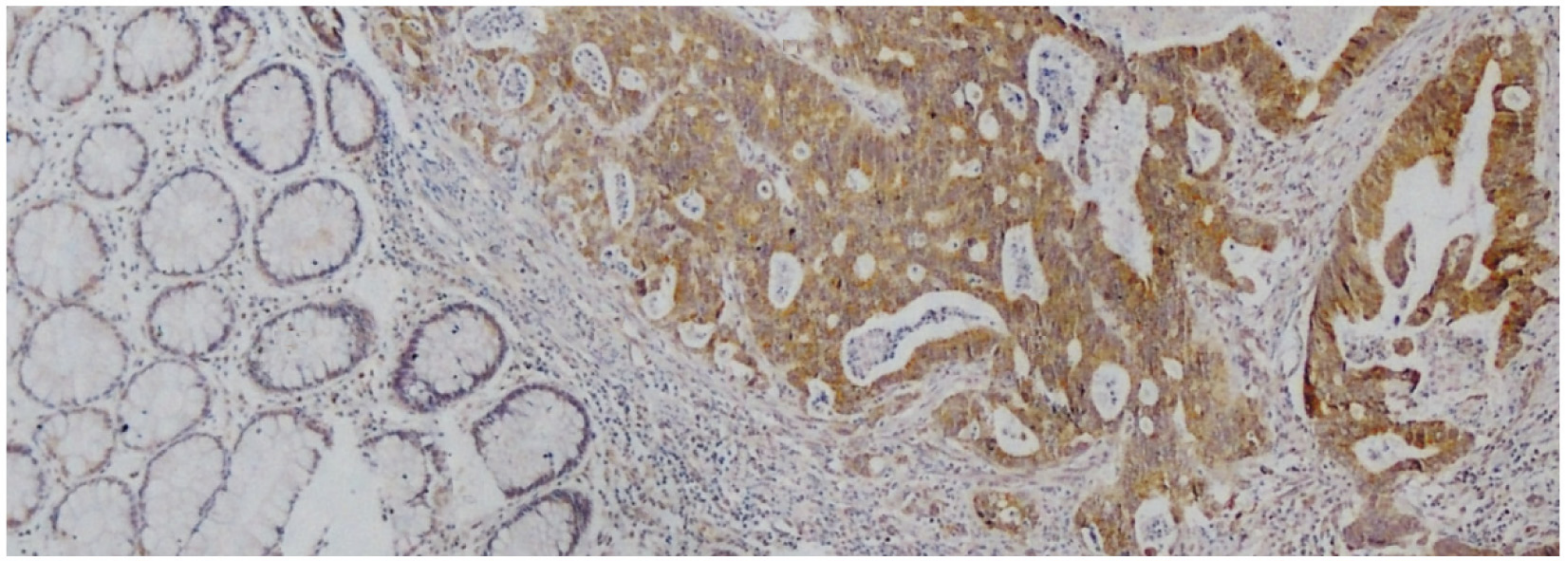
Immunohistochemical analysis of GATA2 expression in colorectal tissues. Representative image indicated strong GATA2 staining in CRC tissue, and negative GATA2 staining in matched noncancerous tissue. Magnification is 100×.

**Fig 5:**
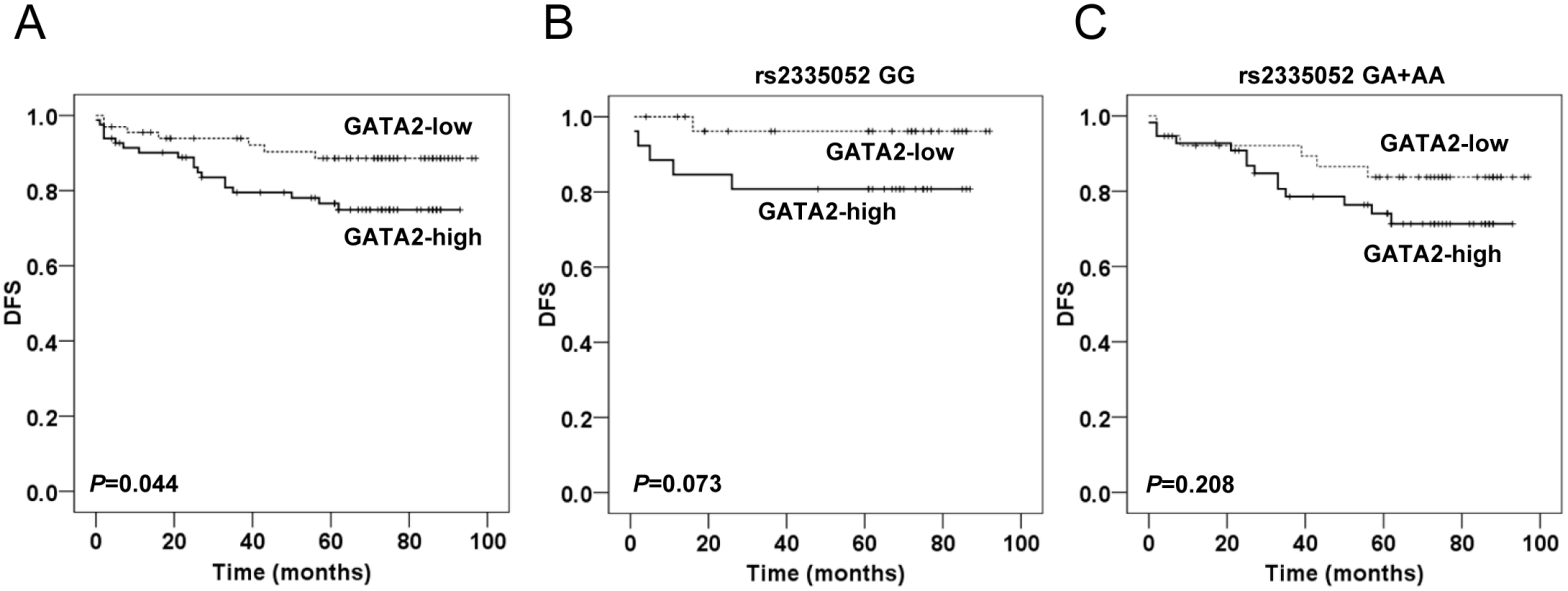
Kaplan-Meier analysis of DFS according to GATA2 expression. **(A)** Kaplan-Meier survival curve showed DFS for patients with GATA2-high tumors versus patients with GATA2-low tumors. Kaplan-Meier survival curves showed DFS for patients stratified by GATA2 rs2335052 GG **(B)**, and GG+AA **(C)** genotypes. The log-rank test was used to calculate *P* values.

The expression of GATA2 was examined in 164 of 180 CRC tissues (Fig 4). High GATA2 expression was detected in 93 out of 164 (56.7%) CRC tissues, compared with 7 out of 97 (7.2%) in matched adjacent noncancerous tissues (*P* < 0.0001). Kaplan-Meier curve for DFS showed that patients with tumors exhibiting high GATA2 expression had worse prognosis, suggesting shorter DFS and earlier disease recurrence (*P* = 0.044, Fig 5A). However, no significant association was observed between GATA2 expression and SNP rs2335052 (*P* = 0.523, Table 1).

Since SNP rs2335052 GG genotype showed a significant protective influence on DFS in the dominant model of inheritance (Fig 2B), for further analysis, patients were divided into two groups according to their rs2335052 genotypes (GG subgroup, GA and AA subgroup). In the rs2335052 GG subgroup, high levels of GATA2 expression showed borderline significant association with shorter DFS, compared with low GATA2 expression (*P* = 0.073, Fig 5B). However, there was no significant association between the levels of GATA2 expression and DFS in the subgroup of patients carrying the rs2335052 GA and AA genotypes (*P* = 0.208, Fig 5C).

## Discussion

In this study, we investigated the association between the GATA2 SNP rs2335052 and the prognosis of CRC patients in a Chinese cohort. Our data revealed that although there was no significant correlation between the rs2335052 genotypes and the expression of GATA2, the rs2335052 GA and AA genotypes were significantly associated with unfavorable prognosis and increased risk of recurrence in patients with CRC, compared with those carrying the GG genotype. Moreover, univariate and multivariate Cox regression analysis demonstrated that the rs2335052 SNP in GATA2 was an independent predictor for poor DFS in CRC patients.

Several previous studies have demonstrated the influence of GATA2 expression levels on the clinical outcome in patients with pediatric acute myeloid leukemia (AML), colorectal, prostate, breast cancer, and hepatocellular carcinoma [14, 16–20]. Except hepatocellular carcinoma [20], high levels of GATA2 expression indicate tumor recurrence, metastasis, or poor survival [14, 16–19]. Besides the protein level of GATA2, the genetic diversity of the GATA2 locus also influences tumorigenesis. Acquired and inherited mutations in GATA2 have been reported to be correlated with myeloid malignancies including AML, and chronic myeloid leukemia (CML) [13–15]. Rearrangement of the GATA2 enhancer to EVI1 causes reduced and monoallelic expression of GATA2, as well as activation of EVI1 in sporadic familial AML/MDS and MonoMac/Emberger symdromes [26]. However, the role of GATA2 genotypes in cancer prognosis remains largely unexplored in solid tumors. Since SNPs locate in the coding and regulatory sequences of GATA2, they may contribute to the regulation of GATA2 expression and function, so we investigated the GATA2 SNP genotypes.

According to the NCBI SNP database, there are several SNPs located at the GATA2 locus. We first sequenced the whole exon of GATA2 in 23 CRC tissues, in order to identify SNPs of potential interest. Our results showed that SNP rs2335052 could be detected in the third exon of GATA2 gene among the 23 CRC tissues, although other genetic variants were absent, probably due to limited sample size (S1 Table). Then, we determined the GATA2 SNP rs2335052 and the expression of GATA2 in a CRC cohort consisting of 180 patients, and investigated the influence of the SNP rs2335052 on patient survival. The results indicated that GATA2 rs2335052 GA and AA genotypes are significantly associated with reduced DFS and recurrence in CRC patients. In addition, the analysis of a replication cohort of 61 CRC patients also suggested that GATA2 SNP rs2335052 was an independent predictor for poor DFS in CRC patients (S2 and S3 Tables, S1 Fig).

Several studies have shown that being a transcription factor, GATA2 acts as a key regulator in many signaling pathways. In KRAS mutant non-small cell lung cancer (NSCLC), GATA2 is involved in the regulation of the proteasome, IL-1-signaling, and Rho-signaling pathways. As a result, GATA2 is indispensable for the survival of NSCLC cells with RAS-pathway mutations [27, 28]. In prostate cancer, GATA2 plays a crucial role in mediating androgen receptor (AR) expression, as well as in site-specific AR recruitment facilitating AR target gene expression [29]. In breast cancer, GATA2 promotes cell proliferation and stimulates AKT phosphorylation by inhibiting PTEN transcription [19]. These raise the possibility of regulating GATA2-involved pathways for cancer therapeutic strategies [27, 28]. Similarly, SNPs that are associated with GATA2 function and clinical outcomes may serve as easily detectable predictors in GATA2-related treatment in the future.

Moreover, as a transcription factor, small genetic alterations in GATA2 may contribute to the regulation of downstream genes. The variant rs2335052 is located within the coding region of GATA2, leading to a substitution of alanine to threonine at codon 164 (A164T) in the third exon. The change of this amino acid is not located at zinc finger regions, which play important roles in GATA2 binding to DNA. To understand the underlying mechanisms involved in the prognostic value of GATA2 SNP rs2335052 on DFS, we predicted the function of GATA2 with amino acid substitute (GATA2-A164T) by Polymorphism Phenotyping v2 (PolyPhen-2), SIFT, and Pmut softwares, which are available via the websites, and determined the transactivation ability of GATA2-A164T by luciferase reporter assays. Although the software-predicted results showed that A164T does not predict potential pathological impact on the function of GATA2 (S2 Fig), the results of luciferase reporter assays suggested that GATA2-A164T reduced the transactivation ability compared to wild type GATA2 (GATA2wt) on the known GATA2-responsive LYL1 promoter [30]. In addition, decreased GATA2-A164T expression was observed in CRC cell lines (S1 File, S4 Table, S3 Fig). These data raised the possibility that GATA2-A164T affect the expression or function of downstream genes which are associated with the survival of CRC patients. Although, in this study, no significant association was found between GATA2 rs2335052 genotype and gene expression in the patient cohort, the decreased expression and reduced transactivation ability of GATA2 A164T, compared with wild type GATA2, indicate that reduced GATA2 expression may contribute to decreased disease-free survival (DFS). Inconsistantly, in this study and our previous study, decreased GATA2 expression was associated with better survival in patients with CRC.

Similarly, many studies reported that although variant genotype may regulate the expression level of that gene, the association between the polymorphisms and survival may be inconsistent with the association between the gene expression and survival [31–34]. For example, patients with wild type AA genotype in ABCC1 rs35628 had longer DFS than patients carrying the G allele. Tumors from carriers of the AA genotype expressed significantly higher ABCC1 than those with the G allele. These data raised the possibility that high expression of ABCC1 may contribute to prolonged survival. In contrast, high expression levels of ABCC1 indicates poor prognosis for breast carcinoma [31, 32]. In addition, patients with VV genotype in NPAS2 rs1053090 conferred a significantly increased risk of death, compared with those with WW and WV genotype. Patients carrying the VV genotype also showed a significantly higher expression level of NPAS2. However, high level of NPAS2 expression was significantly correlated with better DFS [33, 34]. Therefore, other molecular consequences of genetic variation of SNP rs2335052 may exist.

CRC Patients within the same TNM stage may show considerably different clinical outcomes, particularly in stage II disease [6, 7]. The use of adjuvant therapy for stage II CRC patients remains controversial [35–38]. Thus, in order to determine whether SNP rs2335052 in GATA2 could be used to identify patients with a high risk of recurrence so that they may receive further chemotherapy, the role of SNP rs2335052 on DFS and recurrence was evaluated. In the current cohort, among the patients with stage I/II diseases, rs2335052 GA and AA genotypes suggest poor clinical outcome and recurrence, compared with the GG genotype. Our results provided GATA2 SNP rs2335052 as a prognostic factor independent of tumor stages, and raise the possibility to identify patients with higher risk of recurrence and should be treated with adjuvant chemotherapy.

However, there are potential limitations of this study. The cohort in our study is of moderate sample size. Therefore, additional studies with larger independent populations are needed to further validate the association of the GATA2 SNP rs2335052 with the clinical outcome of CRC patients.

## Conclusions

In conclusion, our study demonstrated that SNP rs2335052 in GATA2 is associated with DFS and recurrence of CRC patients. The results of our study suggested that SNP rs2335052 is an independent indicator for predicting the prognosis of CRC in Chinese population. Further studies are needed to validate these findings and clarify the underlying mechanism.

## Supporting Information

S1 Fig Kaplan-Meier analysis of DFS according to GATA2 SNP rs2335052 in another cohort including 61 CRC patients. Kaplan-Meier curves are shown for an additive **(A)**, dominant **(B)**, and recessive model **(C)** of inheritance. The log-rank test was used to calculate *P* values.
(TIF)

S2 Fig Results of the software analysis for alanine to threonine substitution (A164T) of GATA2 SNP rs2335052. A164T did not predict potential pathological impact on the function or structure of GATA2, by using PolyPhen-2 **(A)**, SIFT **(B)**, and Pmut **(C)**, which are available via the websites.
(TIF)

S3 Fig GATA2-A164T reduced the transactivation ability on the LYL1 promoter. **(A)** Western blot analysis of exogenous FLAG-GATA2 expression in RKO cells transfected with plasmid encoding GATA2wt or GATA2-A164T. **(B)** Luciferase reporter assay demonstrated that GATA2-A164T reduced the transactivation ability on the known GATA2-responsive LYL1 promoter, compared with GATA2wt. The data represent the mean ± SD of three independent experiments in triplicate of each sample. In all comparisons, a Student’s t-test was used.
(TIF)

S1 File Materials and Methods. (DOC)

S1 Table All the polymorphisms analyzed in the 23 CRC tissues. (DOCX)

S2 Table Clinicopathological features with respect to determined genotypes of included GATA2 SNP rs2335052 in another cohort including 61 CRC patients. (DOCX)

S3 Table Univariate and multivariate analyses of GATA2 rs2335052 genotypes in 61 CRC patients with respect to DFS. (DOCX)

S4 Table Primer sequences for plasmid construction. (DOCX)
