# Supplementary figures and images for: GATA2 rs2335052 Polymorphism Predicts the Survival of Patients with Colorectal Cancer
Source: PLoS One. 2015 Aug 19;10(8):e0136020. doi: 10.1371/journal.pone.0136020 (PMC4546112; doi:10.1371/journal.pone.0136020)

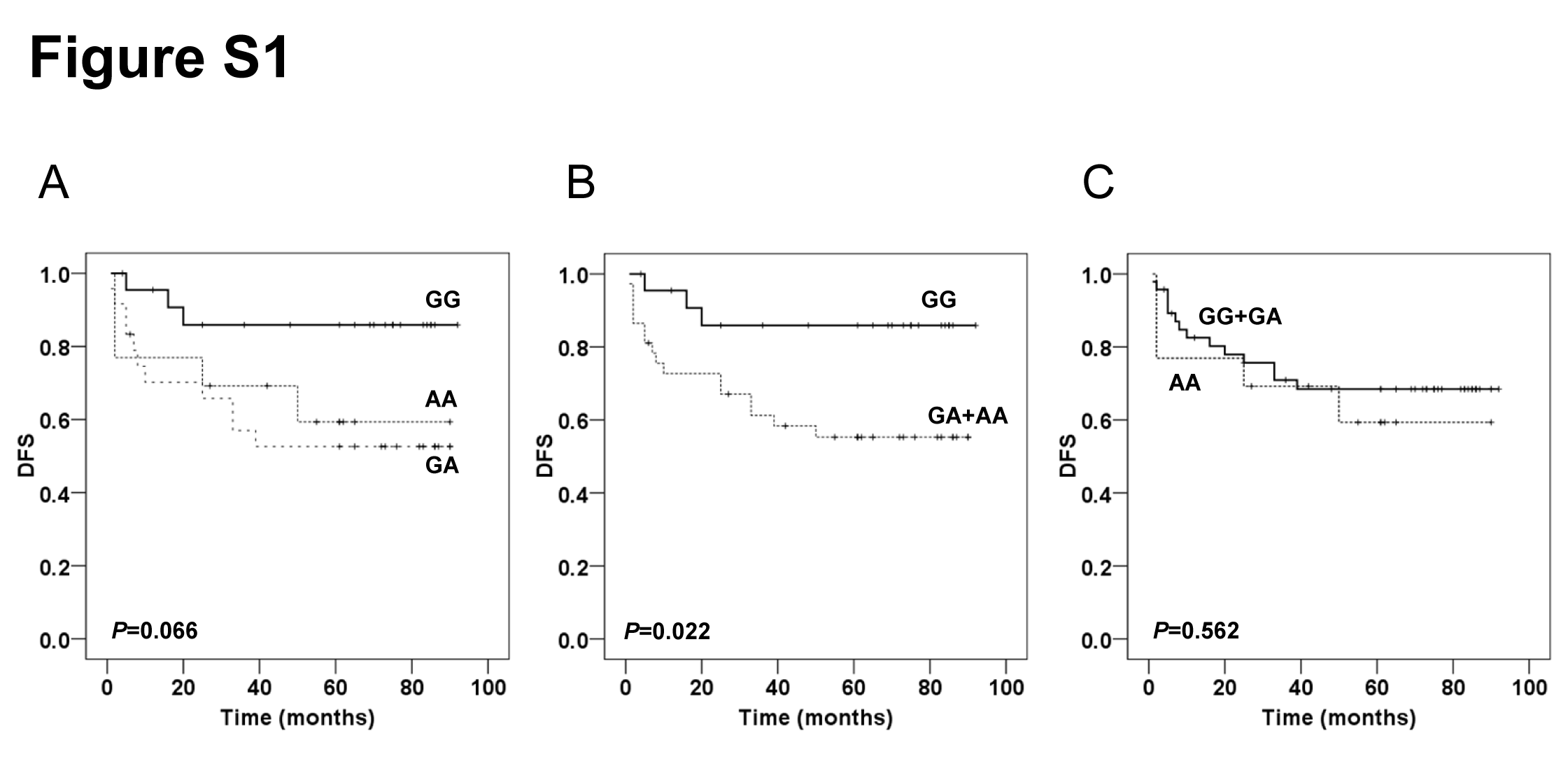

Supplement: S1 Fig — Kaplan-Meier curves are shown for an additive (A), dominant (B), and recessive model (C) of inheritance. The log-rank test was used to calculate P values. (TIF) [file pone.0136020.s001.tif]

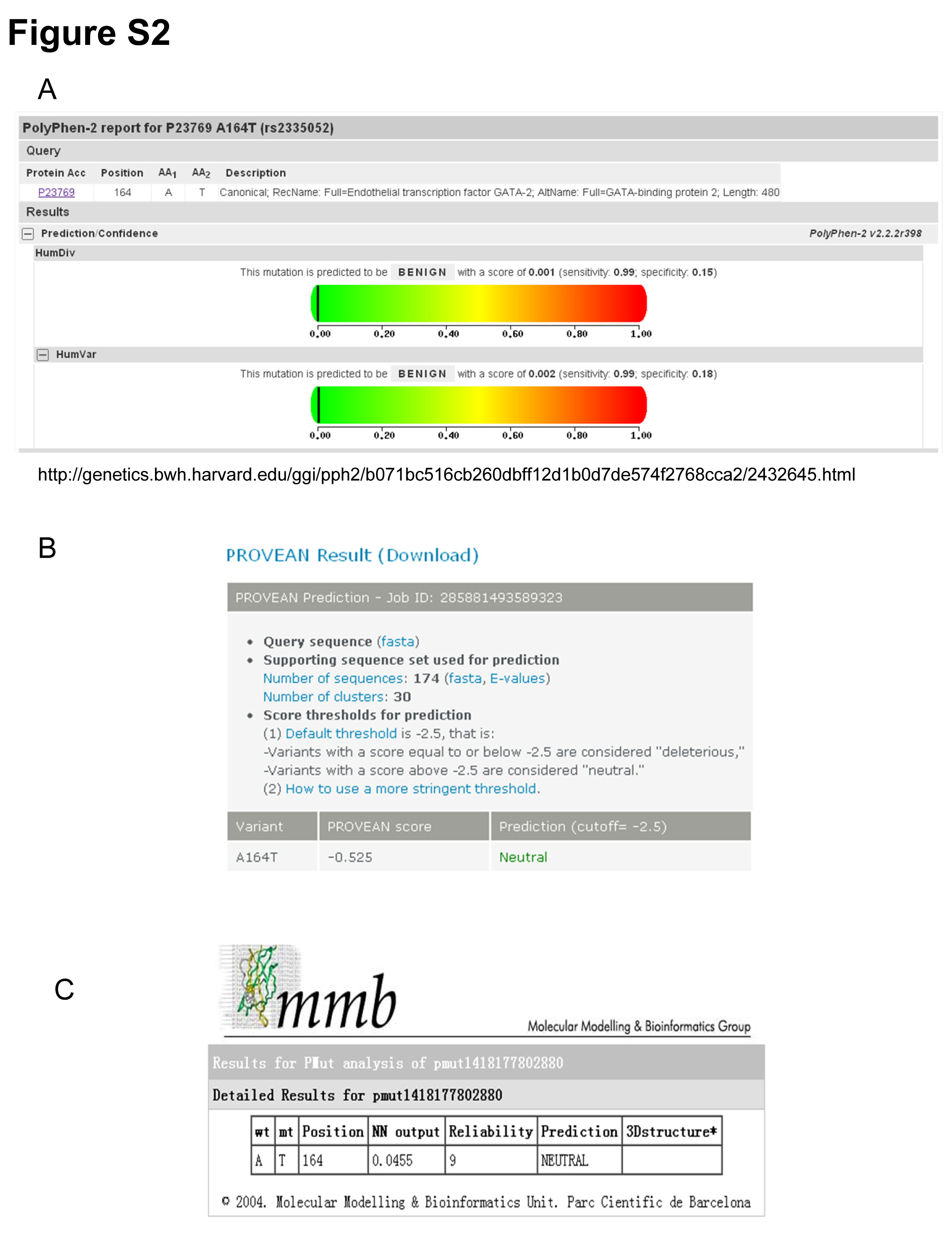

Supplement: S2 Fig — A164T did not predict potential pathological impact on the function or structure of GATA2, by using PolyPhen-2 (A), SIFT (B), and Pmut (C), which are available via the websites. (TIF) [file pone.0136020.s002.tif]

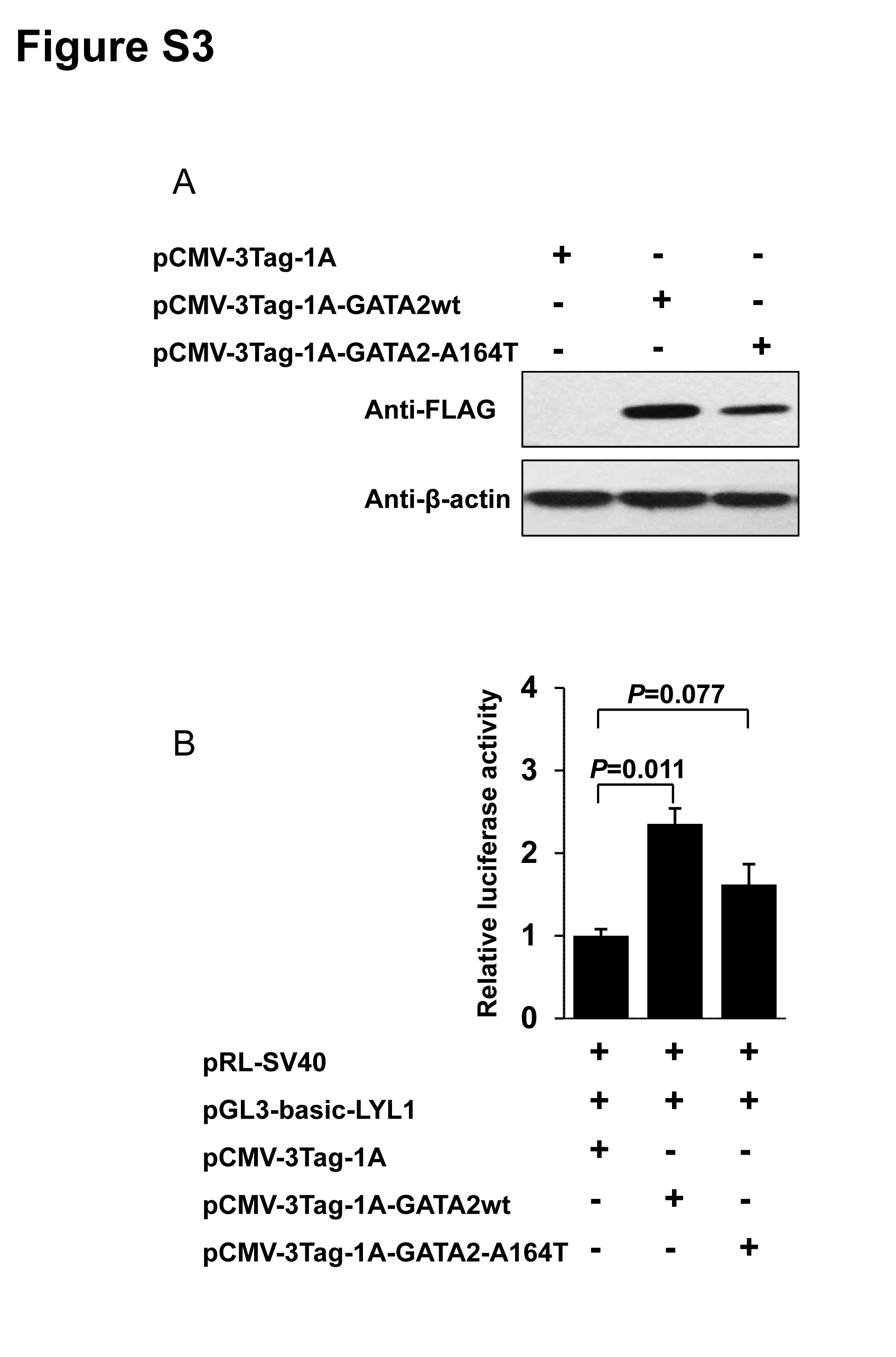

Supplement: S3 Fig — (A) Western blot analysis of exogenous FLAG-GATA2 expression in RKO cells transfected with plasmid encoding GATA2wt or GATA2-A164T. (B) Luciferase reporter assay demonstrated that GATA2-A164T reduced the transactivation ability on the known GATA2-responsive LYL1 promoter, compared with GATA2wt. The data represent the mean ± SD of three independent experiments in triplicate of each sample. In all comparisons, a Student’s t-test was used. (TIF) [file pone.0136020.s003.tif]
