# Supplementary material for: GATA2 rs2335052 Polymorphism Predicts the Survival of Patients with Colorectal Cancer
Source: PLoS One. 2015 Aug 19;10(8):e0136020. doi: 10.1371/journal.pone.0136020 (PMC4546112; doi:10.1371/journal.pone.0136020)
Supplement: S1 File — (DOC) [file pone.0136020.s004.doc]

**Materials and Methods S1**

**Plasmid Construction**

The entire coding sequence of human GATA2 (NM_032638.4) or GATA2-A164T (GATA2 with a substitution of alanine to threonine at codon 164) were obtained by RT–PCR and were cloned into the pCMV-3Tag-1A vector, which contains an N-terminal FLAG tag. For luciferase reporter plasmid, the human LYL1 promoter sequence (NG_027756.1), which include GATA2 binding site, was amplified by PCR from genomic DNA and cloned into the pGL3-Basic vector (Promega, Madison, WI, USA). The sequences of the primers are listed in S4 Table. All of the constructs were verified by DNA sequencing.

**Western blot**

Cells were harvest and total protein (100 μg) isolated from cells were electrophoresed followed by electrotransferring onto a nitrocellulose membrane. The expression of proteins was detected using primary antibody against FLAG M2 (Sigma-Aldrich, St. Louis, MO, USA, F3165) or β-actin (AC-15, Sigma-Aldrich, A-5441). The signal was detected by the ECL Western blot detection kit (Amersham, Little Chalfont, UK).

**Luciferase Reporter Assay**

Cells were seeded in 24-well plates and co-transfected with 300 ng pGL3-Basic-LYL1, 26ng pRL-SV40 plasmid expressing Renilla luciferase, and 500 ng pCMV-3Tag-1A, pCMV-3Tag-1A-GATA2wt, or pCMV-3Tag-1A-GATA2-A164T using Lipofectamine^TM^ 2000 (Invitrogen). Luciferase activity was measured 48 h after transfection using the Dual-Luciferase reporter assay system (Promega) according to the manufacturer’s protocol. Firefly luciferase activity was normalized to Renilla luciferase activity. All assays were performed three times in triplicate. All results were analyzed using Student’s t-test. *P*<0.05 was considered statistically significant.
