# Supplementary material for: GATA2 rs2335052 Polymorphism Predicts the Survival of Patients with Colorectal Cancer
Source: PLoS One. 2015 Aug 19;10(8):e0136020. doi: 10.1371/journal.pone.0136020 (PMC4546112; doi:10.1371/journal.pone.0136020)
Supplement: S1 Table — (DOCX) [file pone.0136020.s005.docx]

**Table S1** All the polymorphisms analyzed in the 23 CRC tissues.

| SNP | Gene Location | Alleles | Case (%) | Primers |
| --- | --- | --- | --- | --- |
| N/A | Exon1 | N/A | 0/23(0) | F:5’-TATACACTGATACCTATGCT-3’ |
|  |  |  |  | R:5'-TATTTTTATTTTTATTTTTG-3' |
| rs17851306 C(Pro)→G(Arg) | Exon2 | C/G | 0/23(0) | F:5’-GCCTCACTCCCCCTTCCT-3’ |
| rs1573858 C(Pro)→G(Pro) |  | C/G | 0/23(0) | R:5’-GCCTGGGTTCTCATCACCA-3’ |
| rs367785289 G(Trp)→T(Cys) |  | G/T | 0/23(0) |  |
| rs372722885 G(Pro)→T(Pro) |  | G/T | 0/23(0) |  |
| **rs2335052  G(Ala)→A(Thr)** | Exon3 | G/A | 15/23(65.22) | F:5’-CCACCCTGATCCTCTCTCTCTTT-3’ |
| rs370750401 G(Asp)→A(Asn) |  | G/A | 0/23(0) | R:5’-TCACAGCTCCCCACCACAA-3’ |
| rs528737073 C( Pro )→G(Ala) |  | C/G | 0/23(0) |  |
| rs142693553 C( Ser )→A(Tyr ) |  | C/A | 0/23(0) |  |
| rs148024280 T(Cys)→C(Arg) | Exon4 | T/C | 0/10(0) | F:5’-GAACTTGCCGGTTAAGCAGG-3’ |
| rs141800945 G(Gly)→A(Ser) |  | G/A | 0/10(0) | R:5’-GCAAAGCGTCTGCAT TTGAA-3 |
| rs367955980 C(Arg)→ G(Gly) |  | C/G | 0/10(0) |  |
| rs376351188 T(Pro)→ C(Pro) |  | T/C | 0/10(0) |  |
| rs370166358 A(Ser)→G(Ser) | Exon5 | A/G | 0/20(0) | F:5’-TTGAT TCCCAGTGAGATTTAGC -3’ |
| rs376360090 T(Ala)→C(Ala) |  | T/C | 0/20(0) | R:5’-CCTCTTGCCTGGCAGCAC-3’ |
| rs143554523 T(Val)→C(Ala) |  | T/C | 0/20(0) |  |
| rs371599112 G(Ala)→C(Ala) |  | G/C | 0/20(0) |  |
| rs148942346 T(lle)→C(Thr) | Exon6 | T/C | 0/23(0) | F: 5’-TGTTGCTGGAGGAAGGAACTG-3’ |
| rs146814201 T(Asn)→C(Asn ) |  | T/C | 0/23(0) | R: 5’-TGTCGGCCT TCGGGA AAT-3’ |
| rs376003468 T(Asn)→C(Asn ) |  | T/C | 0/23(0) |  |
| rs145076941 G(Glu)→A(Glu ) |  | G/A | 0/23(0) |  |
| rs371096438 T(Asn)→ G(Lys) |  | T/G | 0/23(0) |  |
| rs375927513 G(Ser )→A(Asn ) |  | G/A | 0/23(0) |  |
| rs376420351 T(Asn)→C(Asn) |  | T/C | 0/23(0) |  |
| rs374457534 T(Val)→C(Ala) |  | T/C | 0/23(0) |  |
| rs34172218 A(Ala)→G(Ala) |  | A/G | 0/23(0) |  |
| rs146554939 T(Leu)→C(Ser) |  | T/C | 0/23(0) |  |
| rs201155045 C(Thr)→ G(Ser ) |  | C/G | 0/23(0) |  |
| rs1126559 A(Met)→G(Val ) |  | A/G | 0/23(0) |  |
| rs150052821 A(Ser)→C(Ser) |  | A/C | 0/23(0) |  |
| rs370164300A( Arg ) → G(Gly) |  | A/G | 0/23(0) |  |
