# Supplementary material for: GATA2 rs2335052 Polymorphism Predicts the Survival of Patients with Colorectal Cancer
Source: PLoS One. 2015 Aug 19;10(8):e0136020. doi: 10.1371/journal.pone.0136020 (PMC4546112; doi:10.1371/journal.pone.0136020)
Supplement: S2 Table — (DOCX) [file pone.0136020.s006.docx]

**Table S2** Clinicopathological features with respect to determined genotypes of included GATA2 SNP rs2335052 in another cohort including 61 CRC patients.

| Characteristic |  | Cases (%) | GA (%) | GG (%) | AA (%) | P-value |
| --- | --- | --- | --- | --- | --- | --- |
| Age | <60 yr | 24 (39.3) | 12 (50.0) | 5 (21.7) | 7 (50.0) | 0.091 |
|  | ≥60 yr | 37 (60.7) | 12 (50.0) | 18 (78.3) | 7 (50.0) |  |
| Gender | Female | 22 (36.1) | 8 (33.3) | 9 (39.1) | 5 (35.7) | 0.981 |
|  | Male | 39 (63.9) | 16 (66.7) | 14 (60.9) | 9 (64.3) |  |
| Tumor location | Colon | 47 (77.0) | 19 (79.2) | 19 (82.6) | 9 (64.3) | 0.416 |
|  | Rectum | 14 (23.0) | 5 (20.8) | 4 (17.4) | 5 (35.7) |  |
| Tumor size | ≤4cm | 30 (50.0) | 10 (43.5) | 11 (47.8) | 9 (64.3) | 0.454 |
|  | >4cm | 30 (50.0) | 13 (56.5) | 12 (52.2) | 5 (35.7) |  |
|  | Unknown | 1 |  |  |  |  |
| Depth of invasion | T1/T2 | 2 (3.3) | 2 (8.3) | 0 (0.0) | 0 (0.0) | 0.203 |
|  | T3/T4 | 59 (96.7) | 22 (91.7) | 23 (100.0) | 14 (100.0) |  |
| Lymph node involvement | Negative | 35 (57.4) | 15 (62.5) | 15 (65.2) | 5 (35.7) | 0.172 |
|  | Positive | 26 (42.6) | 9 (37.5) | 8 (34.8) | 9 (64.3) |  |
| AJCC Stage | I/II | 32 (52.5) | 13 (54.2) | 15 (65.2) | 4 (28.6) | 0.094 |
|  | III/IV | 29 (47.5) | 11 (45.8) | 8 (34.8) | 10 (71.4) |  |
| Histological type | Adenocarcinoma | 54 (88.5) | 22 (91.7) | 20 (87.0) | 12 (85.7) | 0.820 |
|  | Mucinous | 7 (11.5) | 2 (8.3) | 3 (13.0) | 2 (14.3) |  |
| Tumor grade | Well | 3 (5.6) | 1 (4.5) | 0 (0.0) | 2 (16.7) | 0.342 |
|  | Moderate | 49 (90.7) | 21 (90.9) | 19 (95.0) | 11 (83.3) |  |
|  | Poor | 2 (3.7) | 1 (4.5) | 1 (5.0) | 0 (0.0) |  |
|  | Unknown | 7 |  |  |  |  |
| Chemotherapy | Not received | 24 (39.3) | 7 (29.2) | 9 (39.1) | 8 (57.1) | 0.235 |
|  | Received | 37 (60.7) | 17 (70.8) | 14 (60.9) | 6 (42.9) |  |
| Disease-free survival | Negative | 41 (68.3) | 13 (54.2) | 20 (87.0) | 8 (61.5) | 0.045 |
|  | Positive | 19 (31.7) | 11 (45.8) | 3 (13.0) | 5 (38.5) |  |
|  | Unknown | 1 |  |  |  |  |
| Overall survival | Alive | 38 (63.3) | 17 (70.8) | 16 (69.6) | 5 (38.5) | 0.109 |
|  | Dead | 22 (36.7) | 7 (29.2) | 7 (30.4) | 8 (61.5) |  |
|  | Unknown | 1 |  |  |  |  |

Differences in categorical study variables between genotypes were tested for statistical significance with the Chi-squared test. Tumors were classified according to the guidelines of the American Joint Committee on Cancer (AJCC) staging system.
