# Supplementary material for: GATA2 rs2335052 Polymorphism Predicts the Survival of Patients with Colorectal Cancer
Source: PLoS One. 2015 Aug 19;10(8):e0136020. doi: 10.1371/journal.pone.0136020 (PMC4546112; doi:10.1371/journal.pone.0136020)
Supplement: S3 Table — (DOCX) [file pone.0136020.s007.docx]

**Table S3** Univariate and multivariate analyses of GATA2 rs2335052 genotypes in 61 CRC patients with respect to DFS.

| Variables | Univariate |  |  | | Multivariate |  |  | |
| --- | --- | --- | --- | --- | --- | --- | --- | --- |
|  | HR | 95%CI | | *P* | HR | 95%CI | | *P* |
| Age (≥60 yr vs <60 yr) | 1.086 | 0.436-2.702 | | 0.860 | 0.740 | 0.252-2.172 | | 0.584 |
| Gender (Male vs Female) | 1.150 | 0.437-3.028 | | 0.777 | 1.421 | 0.442-4.574 | | 0.556 |
| Tumor location (Rectum vs Colon ) | 0.818 | 0.271-2.469 | | 0.722 | 0.533 | 0.136-2.094 | | 0.368 |
| Tumor size (>4cm vs ≤4cm) | 1.425 | 0.573-3.545 | | 0.446 | 0.450 | 0.127-1.601 | | 0.217 |
| TNM stage (III/IV vs I/II) | 2.673 | 1.048-6.815 | | **0.040** | 2.704 | 0.876-8.346 | | 0.084 |
| Tumor differentiation (Poor/moderate vs Well) | 3.546 | 2.070-6.072 | | **<0.0001** | 8.863 | 3.207-24.496 | | **<0.0001** |
| Chemotherapy (Received vs Not received) | 0.975 | 0.348-2.480 | | 0.958 | 2.365 | 0.598-9.348 | | 0.220 |
| GATA2 SNP (GA/AA vs GG) | 3.760 | 1.095-12.909 | | **0.035** | 5.164 | 1.184-22.527 | | **0.029** |
|  |  |  |  | |  |  |  | |

*HR* hazard ratio, *CI* confidence interval, *P* values in bold were statistically significant
