# Supplementary material for: GATA2 rs2335052 Polymorphism Predicts the Survival of Patients with Colorectal Cancer
Source: PLoS One. 2015 Aug 19;10(8):e0136020. doi: 10.1371/journal.pone.0136020 (PMC4546112; doi:10.1371/journal.pone.0136020)
Supplement: S4 Table — (DOCX) [file pone.0136020.s008.docx]

**Table S4** Primer sequences for plasmid construction.

| Primer Name | Primer Sequence |
| --- | --- |
| GATA2wt-F | CCGGAATTCATGGAGGTGGCGCCCGAGCAGCCGCGCTG |
| GATA2wt-R | CCGCTCGAGCTAGCCCATGGCGGTCACCATGCTGGAC |
| GATA2-A164T-F | GCGGGAGCTCAGTGGCCTCCCTCACCCCTACAGCAACC |
| GATA2-A164T-R | CCGCTCGAGCTAGCCCATGGCGGTCACCATGCTGGAC |
| LYL1 promoter-F | GCGGGAGCTCTATGTGATCCTGTAGCCAAGAG |
| LYL1 promoter-R | CCGCTCGAGCCCAGCACTGTTCTTGCAGCCT |
